# Natural language processing for mental health interventions: a systematic review and research framework

**DOI:** 10.1038/s41398-023-02592-2

**Published:** 2023-10-06

**Authors:** Matteo Malgaroli, Thomas D. Hull, James M. Zech, Tim Althoff

**Affiliations:** 1 Department of Psychiatry, New York University, Grossman School of Medicine, New York, NY 10016 USA; 2 Talkspace, New York, NY 10025 USA; 3 Department of Psychology, Florida State University, Tallahassee, FL 32306 USA; 4 Department of Computer Science, University of Washington, Seattle, WA 98195 USA

**Keywords:** Psychiatric disorders, Psychology

## Abstract

Neuropsychiatric disorders pose a high societal cost, but their treatment is hindered by lack of objective outcomes and fidelity metrics. AI technologies and specifically Natural Language Processing (NLP) have emerged as tools to study mental health interventions (MHI) at the level of their constituent conversations. However, NLP’s potential to address clinical and research challenges remains unclear. We therefore conducted a pre-registered systematic review of NLP-MHI studies using PRISMA guidelines (osf.io/s52jh) to evaluate their models, clinical applications, and to identify biases and gaps. Candidate studies (n = 19,756), including peer-reviewed AI conference manuscripts, were collected up to January 2023 through PubMed, PsycINFO, Scopus, Google Scholar, and ArXiv. A total of 102 articles were included to investigate their computational characteristics (NLP algorithms, audio features, machine learning pipelines, outcome metrics), clinical characteristics (clinical ground truths, study samples, clinical focus), and limitations. Results indicate a rapid growth of NLP MHI studies since 2019, characterized by increased sample sizes and use of large language models. Digital health platforms were the largest providers of MHI data. Ground truth for supervised learning models was based on clinician ratings (*n* = 31), patient self-report (*n* = 29) and annotations by raters (*n* = 26). Text-based features contributed more to model accuracy than audio markers. Patients’ clinical presentation (*n* = 34), response to intervention (*n* = 11), intervention monitoring (*n* = 20), providers’ characteristics (*n* = 12), relational dynamics (*n* = 14), and data preparation (*n* = 4) were commonly investigated clinical categories. Limitations of reviewed studies included lack of linguistic diversity, limited reproducibility, and population bias. A research framework is developed and validated (NLPxMHI) to assist computational and clinical researchers in addressing the remaining gaps in applying NLP to MHI, with the goal of improving clinical utility, data access, and fairness.

## Introduction

Neuropsychiatric disorders including depression and anxiety are the leading cause of disability in the world [1]. The sequelae to poor mental health burden healthcare systems [2], predominantly affect minorities and lower socioeconomic groups [3], and impose economic losses estimated to reach 6 trillion dollars a year by 2030 [4]. Mental Health Interventions (MHI) can be an effective solution for promoting wellbeing [5]. Numerous MHIs have been shown to be effective, including psychosocial, behavioral, pharmacological, and telemedicine [6–8]. Despite their strengths, MHIs suffer from systemic issues that limit their efficacy and ability to meet increasing demand [9, 10]. The first is the lack of objective and easily administered diagnostics, which burden an already scarce clinical workforce [11] with diagnostic methods that require extensive training. A second is variable treatment quality [12]. Widespread dissemination of MHIs has shown reduced effect sizes [13], not readily addressable through supervision and current quality assurance practices [14–16]. The third is too few clinicians [11], particularly in rural areas [17] and developing countries [18], due to many factors, including the high cost of training [19]. As a result, the quality of MHI remains low [14], highlighting opportunities to research, develop and deploy tools that facilitate diagnostic and treatment processes.

Recent innovations in the fields of Artificial Intelligence (AI) and machine learning [20] offer options for addressing MHI challenges. Technological and algorithmic solutions are being developed in many healthcare fields including radiology [21], oncology [22], ophthalmology [23], emergency medicine [24], and of particular interest here, mental health [25]. An especially relevant branch of AI is Natural Language Processing (NLP) [26], which enables the representation, analysis, and generation of large corpora of language data. NLP makes the quantitative study of unstructured free-text (e.g., conversation transcripts and medical records) possible by rendering words into numeric and graphical representations [27]. MHIs rely on linguistic exchanges and so are well suited for NLP analysis that can specify aspects of the interaction at utterance-level detail for extremely large numbers of individuals, a feat previously impossible [28]. Typically unexamined characteristics of providers and patients are also amenable to analysis with NLP [29] (Box 1). NLP for MHI began with pre-packaged software tools [30], followed by more computationally intense deep neural networks [31], particularly large language models (i.e., attention-based architectures such as Transformers) [32], and other methods for identifying meaningful trends in large amounts of data. The diffusion of digital health platforms has made these types of data more readily available [33]. These data make it possible to study treatment fidelity [33], estimate patient outcomes [34], identify treatment components [35], evaluate therapeutic alliance [36], and gauge suicide risk [37] in a transformative way, sufficient to generate anticipation and apprehension regarding conversational agents [38]. Lastly, NLP has been applied to mental health-relevant contexts outside of MHI including social media [39] and electronic health records [40].

While these studies demonstrate NLP’s research potential, questions remain about its impact on clinical practice. A significant limiting factor is the current separation between two communities of expertise: clinical science and computer science. Clinical researchers possess domain knowledge on MHI but have difficulty keeping up with the rapid advances in NLP. The clearest reflection of this separation is the continued reliance of clinical researchers on traditional expert-based dictionary methods [30] versus the ongoing state-of-the-art developments in large language models within computer science [32]. Accordingly, while prior reviews provided insights into the growing role of machine learning in mental health [25, 41], they did not include peer-reviewed manuscripts from AI conferences where many advances in NLP are reported. In addition, NLP pipelines were not deconstructed into algorithmic components, limiting the ability to identify distinctive model features. Meanwhile, computer scientists and computational linguists are driving developments in NLP that, while methodologically advanced, are typically limited in their applicability to clinical service provision.

We therefore conducted a systematic review of NLP studies for mental health interventions, examining their algorithmic and clinical characteristics to promote the intersection between computer and clinical science. Our aim was threefold: 1) classify NLP methods deployed to study MHI; 2) identify clinical domains and use them to aggregate NLP findings; 3) identify limitations of current NLP applications to recommend solutions. We examined each manuscript for clinical components (setting, aims, transcript source, clinical measures, ground truths and raters) and key features of the NLP pipeline (linguistic representations and features, classification models, validation methods, and software packages). Finally, we explored common areas, biases, and gaps in the current NLP applications for MHI, and proposed a research framework to address these limitations.

Box 1 Overview and glossary of terms for Natural Language Processing (NLP)

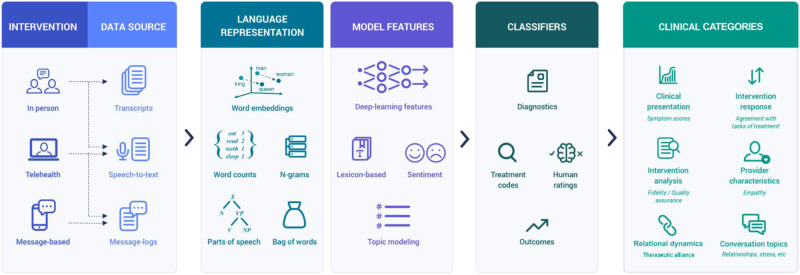

Language Representation.

*Word Embeddings:* Words are mapped to a numeric vector space by an algorithm (e.g., word2vec) based on how they are used and their most frequent neighbor words in a large text dataset. Words have similar values when they co-occur in the same contexts, indicating a shared meaning.

*Word counts (Unigrams):* Single words analyzed based on their frequency.

*N-grams:* Language model consisting of sequences of *n*-number of words, to capture word context (e.g., the bigram “*not depressed*”).

*Part-of-Speech:* Label words by their grammatical and syntactic functions.

*Bag-of-Words/TF-IDF:* Proportional frequency of words or *n*-grams to identify unique features of a text.

Model Features.

*Deep Learning (DL) Features:* DL algorithms are differentiated by number of layers, complexity, and model parameters. Language models are trained using large amounts of text data (e.g., all of Wikipedia), removing random words in a sentence, and learning to fill in the blank. This results in probabilistic models of language that can both interpret and produce text. Transformer architectures (e.g., BERT) also have attention mechanisms to help maintain context connections between distant words. Specific features for clinical tasks are generated by fine-tuning language models on domain-specific datasets.

*Topic Modeling:* Extracts and clusters common topics emerging in a text.

*Lexicon Features:* Matching text to a predefined word list made by human experts.

*Sentiment:* Matching text to emotions. Performed through dictionary methods, human raters, or pre-trained models.

Classifiers (model output)

*Supervised models:* Identify a category or outcome (e.g., diagnosis) after training on a dataset with examples. Human-labeled cases are known as the model’s ‘ground truth’ and performance is measured against match with ground truth labels.

*Unsupervised models:* Derive features from a dataset based on the distribution.

## Methods

### Search protocol and eligibility

The systematic review followed the Preferred Reporting Items for Systematic Reviews and Meta-Analyses (PRISMA) guidelines. The review was pre-registered, its protocol published with the Open Science Framework (osf.io/s52jh). The review focused on NLP for *human-to-human* Mental Health Interventions (MHI), defined as psychosocial, behavioral, and pharmacological interventions aimed at improving and/or assessing mental health (e.g., psychotherapy, patient assessment, psychiatric treatment, crisis counseling, etc.). We excluded studies focused solely on *human-computer* MHI (i.e., conversational agents, chatbots) given lingering questions related to their quality [38] and acceptability [42] relative to human providers. We also excluded social media and medical record studies as they do not directly focus on intervention data, despite offering important auxiliary avenues to study MHI. Studies were systematically searched, screened, and selected for inclusion through the Pubmed, PsycINFO, and Scopus databases. In addition, a search of peer-reviewed AI conferences (e.g., Association for Computational Linguistics, NeurIPS, Empirical Methods in NLP, etc.) was conducted through ArXiv and Google Scholar. The search was first performed on August 1, 2021, and then updated with a second search on January 8, 2023. Additional manuscripts were manually included during the review process based on reviewers’ suggestions, if aligning with MHI broadly defined (e.g., clinical diagnostics) and meeting study eligibility. Search string queries are detailed in the supplementary materials.

### Eligibility and selection of articles

To be included, an article must have met five criteria: (1) be an original empirical study; (2) written in English; (3) vetted through peer-review; (4) focused on MHI; and (5) analyzed text data that was gathered from MHI (e.g., transcripts, message logs). Several exclusion criteria were also defined: (a) study of human-computer interventions; (b) text-based data not derived from human-to-human interactions (i.e., medical records, clinician notes); (c) social media platform content (e.g., Reddit); (d) population other than adults (18+); (e) did not analyze data using NLP; or (f) was a book chapter, editorial article, or commentary. Candidate manuscripts were evaluated against the inclusion and exclusion criteria initially based on their abstract and then on the full-text independently by two authors (JMZ and MM), who also assessed study focus and extracted data from the full text. Disagreement on the inclusion of an article or its clinical categorization was discussed with all the authors following full-text review. When more than one publication by the same authors used the same study aim and dataset, only the study with the most technical information and advanced model was included, with others classified as a duplicate and removed. Reasons for exclusion were recorded.

### Data extraction

Studies that met criteria were further assessed to extract clinical and computational characteristics.

#### Setting and data

The MHI used to generate the data for NLP analyses. Treatment modality, digital platforms, clinical dataset and text corpora were identified.

#### Study focus

Goal of the study, and whether the study primarily examined conversational data from patients, providers, or from their interaction. Moreover, we assessed which aspect of MHI was the primary focus of the NLP analysis.

#### Ground truth

How the concepts of interest were operationalized in each study (e.g., measuring depression as PHQ-9 scores). Information on raters/coders, agreement metrics, training and evaluation procedures were noted where present. Information on ground truth was identified from study manuscripts and first order data source citations.

#### Natural language processing components

We extracted the most important components of the NLP model, including acoustic features for models that analyzed audio data, along with the software and packages used to generate them.

#### Classification model and performance

Where multiple algorithms were used, we reported the best performing model and its metrics, and when human and algorithmic performance was compared.

#### Reproducibility

Information on whether findings were replicated using an external sample separated from the one used for algorithm training, interpretability (e.g., ablation experiments), as well as if a study shared its data or analytic code.

#### Limitations and biases

A formal assessment of the risk of bias was not feasible in the examined literature due to the heterogeneity of study type, clinical outcomes, and statistical learning objectives used. Emerging limitations of the reviewed articles were appraised based on extracted data. We assessed possible selection bias by examining available information on samples and language of text data. Detection bias was assessed through information on ground truth and inter-rater reliability, and availability of shared evaluation metrics. We also examined availability of open data, open code, and for classification algorithms use of external validation samples.

## Results

**Fig. 1:**
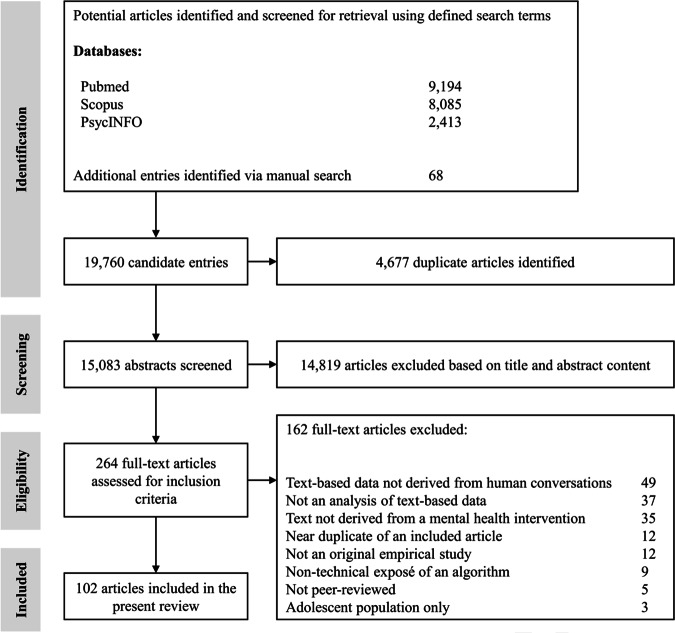
PRISMA flow diagram.

The initial literature screen delivered 19,756 candidate studies. After 4677 duplicate entries were removed, 15,078 abstracts were screened against inclusion criteria. Of these, 14,819 articles were excluded based on content, leaving 259 entries warranting full-text assessment. The screening process is reported in Fig. 1, with the final sample consisting of 102 studies (Table 1).

**Table 1:** Summary of included studies.






*BASIS-24* Behavior and Symptom Identification Scale, *BERT* Bidirectional Encoder Representations from Transformers, *BiLSTM* Bidirectional Long-Short Term Memory, *CBT* Cognitive Behavioral Therapy, *CESD* Center for Epidemiological Studies—Depression scale, *CNN* Convolutional Neural Network, *COPE* Coping Orientation to Problems Experienced, *COVAREP* Collaborative Voice Analysis Repository, *DAAP* Discourse Attributes Analysis Program, *DSM-5* Diagnostic & Statistical Manual of Mental Disorders, Fifth Edition, ELMo Embeddings from Language Model, *GAD-7* General Anxiety Disorder-7, *GloVE* Global Vectors for Word Representation, *GPT-2* Generative Pre-trained Transformer 2, *LIME* Local interpretable model-agnostic explanations, *LIWC* Linguistic inquiry word count, *LSTM* Long-Short Term Memory, *MAE* Mean average error, *MALLET* Machine Learning for LanguagE Toolkit, *MentalBERT* a BERT pre-trained on mental health conversations, *MSE* Mean Squared Error, *NLTK* Natural Language Tool Kit, *PANSS-8* Positive and Negative Syndrome Scale, *PCL-5* PTSD Checklist for DSM-5, *PHQ-9* Patient Health Questionnaire-9, *PSOMS* Positive States of Mind Scale, *PTGI* Posttraumatic Growth Inventory, *PTSD* Post-traumatic stress disorder, *RMSE* Root mean squared error, *RNN* Recurrent Neural Network, *RoBERTa* Robustly Optimized BERT Pre-training Approach, *ROC AUC* Receiver operating characteristic area under the curve, *SF-12* Short Form Survey, *SVM* Support Vector Machine, *SWEMWS* Short Warwick-Edinburgh Mental Well-Being Scale, *TF-IDF* Term frequency—inverse document frequency, *VADER* Valence Aware Dictionary and sEntiment Reasoner, *YAP* Yet Another (natural language) Parser. NLP language representations and model features are associated with their respective software/algorithm by the following symbols: acoustic features, bag of words and TF-IDF, deep learning, lexicon, part-of-speech, sentiment analysis, speech-to-text, topic modeling, word embeddings.

### Study characteristics

#### Publication year

Results indicate a growth of NLP for MHI applications, with the first study appearing in 2010 and the majority being published between 2020–2022 (53.9%, *n* = 55). The median year of publication was 2020 (IQR = 2018–2021), a trend consistent with NLP advancements [32].

#### Setting and data

The majority of interventions consisted of synchronous therapy (53.9%, *n* = 55), with Motivational Interviewing as the most reported therapy modality (*n* = 20). These studies primarily involved face-to-face randomized controlled trials, traditional treatments, and collected therapy corpora (e.g., Alexander Street Corpus). Transcripts of clinical assessments, interviews, and structured tasks were another important source of textual data (20.6%, *n* = 21), elicited through the use of standardized prompts and questions. While most face-to-face studies used text data from manual transcripts, 18 studies used machine-transcription generated from audio sources [36, 43–59]. Online message-based interventions were the second largest setting (22.6%, *n* = 23), with text-data consisting of anonymized conversation logs between providers and patients. Sample sizes increased from less than 100 therapy transcripts [45, 60, 61] to over 100,000 [34, 62–64], with studies analyzing more than one million conversations [65, 66].

#### Ground truth

Clinicians provided ground truth ratings in the form of diagnoses, assessments, or suicide risk for 31 studies. Patients provided ground truth for 29 studies, through self-report measures of symptoms and functioning (*n* = 22), intervention feedback, and treatment alliance ratings. Students (*n* = 9), researchers (*n* = 6), crowd-workers (*n* = 3), and other raters (*n* = 26) provided treatment annotations and emotion/sentiment analysis. As the modal intervention, Motivational Interviewing Skills Codes (MISC) [67] annotations were the most prevalent source of provider/patient information. Thirty-two studies provided information on rater/coder agreement, with adequate inter-rater reliability across studies for frequent and aggregated codes. Only 20 studies provided information on the raters’ training or selection, with Sharma et al., describing in detail an interactive training consisting of instructions, supervision, evaluation, and final selection [62]. Combined human and deep-learning-based approaches were also explored as an alternative to producing a large amount of treatment-related labels. In particular, Ewbank and collegues [34, 35] used a hybrid approach to generate ground truth: human raters annotated a portion of sessions and an annotation model was based on their inputs to label a larger number of sessions.

### Natural language processing and machine learning components

**Fig. 2:**
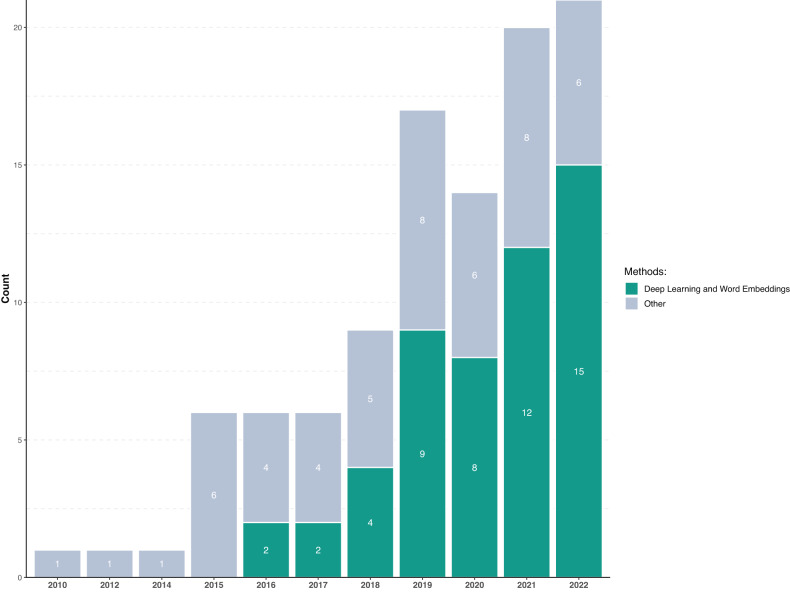
Number of Articles Published per Year.

Multiple NLP approaches emerged, characterized by differences in how conversations were transformed into machine-readable inputs (linguistic representations) and analyzed (linguistic features). Linguistic features, acoustic features, raw language representations (e.g., tf-idf), and characteristics of interest were then used as inputs for algorithmic classification and prediction. Methods used mirrored the development of NLP tools through time (Fig. 2).

#### Language representation

The majority of studies (*n* = 53) tabulated the frequency of individual words through the use of lexicons or dictionaries. Forty-three studies (42.6%) used n-grams for language representation due to their simplicity and interpretability. Bag of Words and Term Frequency-Inverse Document Frequency (TF-IDF) were used by 30 studies to model word frequencies directly for classification purposes [37].

After raw word counts, Word Embeddings were the most commonly utilized language representation (*n* = 49, 48%), owing to its advantages for performing analytic operations. Lower-dimensional embeddings were primarily generated using word2vec and GloVe algorithms. With recent advances in deep learning, more sophisticated Transformer architectures (e.g., RoBERTa) produced contextualized embeddings, where the representation of a word or token depends on its surrounding context.

#### Model features

The most common linguistic features were based on lexicons (*n* = 43) computing the frequency of words by their membership in categories designed by domain experts. This approach is exemplified by software such as LIWC [30], and owes its diffusion in clinical research to its ease of use and low technological requirements. Another prevalent NLP task was sentiment analysis (*n* = 32), which generated feature scores for emotions (e.g., joy, annoyance) that are derived from lexicon-based methods and pre-trained models (e.g., VADER). Topic modeling (*n* = 16) also emerged as a widely used approach to identify common themes across clinical transcripts.

#### Deep learning features

More recent technological developments saw the rise of features based on deep neural networks (*n* = 40). The adoption of large language models grew in parallel with increases in computational power, the development of dedicated code libraries (e.g., Pytorch and Tensorflow), and increased availability of large MHI corpora (Fig. 2). Transformer models were the most used language models given their ability to generate contextually-meaningful linguistic features from sequences of text through the use of attention mechanisms, and to study the flow of individual talk turns [68], as well as its effects on overall session estimates [48].

Models deployed include BERT and its derivatives (e.g., RoBERTa, DistillBERT), sequence-to-sequence models (e.g., BART), architectures for longer documents (e.g., Longformer), and generative models (e.g., GPT-2). Although requiring massive text corpora to initially train on masked language, language models build linguistic representations that can then be fine-tuned to downstream clinical tasks [69]. Applications examined include fine-tuning BERT for domain adaptation to mental health language (MentalBERT) [70], for sentiment analysis via transfer learning (e.g., using the GoEmotions corpus) [71], and detection of topics [72]. Generative language models were used for revising interventions [73], session summarizations [74], or data augmentation for model training [70].

#### Acoustic features

Beyond the use of speech-to-text transcripts, 16 studies examined acoustic characteristics emerging from the speech of patients and providers [43, 49, 52, 54, 57–60, 75–82]. The extraction of acoustic features from recordings was done primarily using Praat and Kaldi. Engineered features of interest included voice pitch, frequency, loudness, formants quality, and speech turn statistics. Three studies merged linguistic and acoustic representations into deep multimodal architectures [57, 77, 80]. The addition of acoustic features to the analysis of linguistic features increased model accuracy, with the exception of one study where acoustics worsened model performance compared to linguistic features only [57]. Model ablation studies indicated that, when examined separately, text-based linguistic features contributed more to model accuracy than speech-based acoustics features [57, 77, 78, 80].

### Clinical research categories

**Fig. 3:**
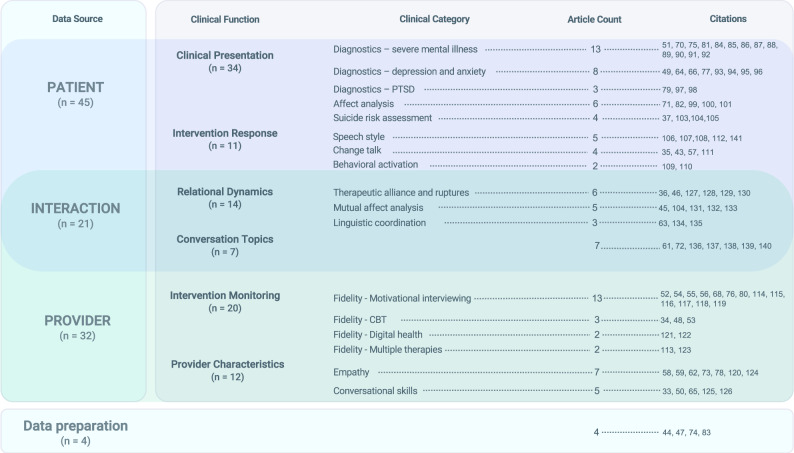
Clinical research categories of reviewed manuscripts.

Three primary sources of data emerged from the examined studies: conversational data from patients (*n* = 45), another set from providers (*n* = 32), and a third set from patient-provider interactions (*n* = 21). In addition, four studies focused on improving NLP data pipelines [47, 74, 44, 83] (Fig. 3). Each of the three data sources were further divided into two subgroups according to study aims. The resulting six clinical categories are discussed further below and composed the central concepts of the integrative framework presented in the discussion.

#### Patient analysis (n = 45)

##### Clinical presentation (*n* = 34)

These studies assessed clinical characteristics evident in transcripts grounded in diagnostic ratings obtained by providers and self-reported symptoms from patients. The premise for these applications is the effect of neuropsychiatric disorders on speech (e.g., neologisms in schizophrenia), sentiment, and content (e.g., worry in anxiety) [29] that act as language-based markers of psychopathology. *Serious Mental Illness (SMI).* Eleven SMI applications used NLP markers to identify psychosis [51, 81, 84, 85] and bipolar [86] diagnoses (Accuracy 0.70 and 0.85), monitor symptoms [75, 87], detect psychotic episodes (*F*_1_ = 0.99) [88] and psychosis onset (AUC = 0.72) [89]. Negative symptoms [87, 90] and cognitive distortions [70] for SMI were detected using linguistic features, including connectedness emerging from graph analytics [90, 91]. Associations between linguistic features and neuroimaging were also examined [91, 92]. *Depression and Anxiety*. Examination of linguistic features showed that lexical diversity [66], the use of more affective language [93] and negative emotions [93, 94] are markers of depression and anxiety [49], and can be used to predict outcomes (QIDS-16 scores, Accuracy = 0.85) [95]. Sentence embeddings [77], n-grams and topics [96] were also used to assess depression and anxiety severity. In addition, linguistic features were able to detect symptoms beyond those typically captured by diagnostic screenings [64]. *Post-Traumatic Stress Disorder* *(PTSD).* Three studies focused on analyzing open-ended trauma narrative to accurately identify PTSD diagnosis [97] and symptom trajectories [98]. Of note, linguistic features from narratives collected one month after life-threatening traumatic events were shown to be predictive of future PTSD (AUC = 0.90) [79]. *Affect Analysis.* Six manuscripts focused on the automatic examination of affect, a component of clinical mental status evaluations. These studies examined emotions at the session- and utterance-level [82], emotional involvement (e.g., warmth) [99], and negativity [100], and emotional distress including exposure therapy hotspots [60]. Sentiment analysis performed similarly to human raters (Cohen’s *K* = 0.58) [101]. Across studies, the latest Transformer-based models were shown to capture emotional valiance [102] and associations with symptom ratings more accurately than other language features [71]. *Suicide Risk.* Another area of clinical interest was suicidality assessment (*n* = 4). While one study focused on lifetime history of suicidality [103], the majority used NLP to assess intentions of suicide or self-harm endorsed during interventions [37, 104, 105], one with sufficient accuracy to be deployed in a clinical setting (AUC = 0.83) [37].

##### Intervention response (*n* = 11)

Eleven studies examined linguistic markers of patient response related to treatment administration [106], outcome [107, 108], patient activation [109, 110], and between-session fluctuation of symptoms [108]. One study identified linguistic markers of behavioral activation in the treatment of depression of 10,000 patients (PHQ-9 scores; R2 = 75.5%) [109]. Three studies captured within-session responses to MHI by examining patients’ responses to provider interventions at *utterance-level* interactions [43, 57, 111]. Of note, Nook et al. showed that clustering a sample of 6,229 patients based on linguistic distance captured differences both in symptoms severity and treatment outcomes [112].

#### Provider analysis (n = 32)

##### Intervention monitoring (*n* = 20)

Most provider analyses focused on monitoring treatment fidelity. These studies segmented interventions into utterance-level elements based on treatment protocols. The majority of treatment fidelity studies examined adherence to Motivational Interviewing (MI) in clinical trial and outpatient settings [52, 54–56, 76, 113–120], with Flemotomos et al. also implementing automated MI fidelity evaluation in practice [52]. Taking advantage of the generative properties of Transformer models, Cao et al. [116]. designed a system that identified MI interventions (MISC codes; *F*_1_ = 0.65) and then forecasted the most likely upcoming intervention (based on the session’s history), with the goal of guiding providers. Other treatment fidelity studies examined the fidelity of Cognitive Behavioral Therapy (CBT) [34, 35, 53, 48] and digital health [121, 122] interventions, with two examining dialogue acts distinguishing different psychotherapy approaches [113, 123]. Treatment fidelity studies primarily relied on human annotators to produce session-level behavioral codes to then train treatment segmentation models. These codes describe the structure of a session compared to the treatment’s typical structure, which does not directly provide evidence for the effectiveness of specific interventions. A demonstration of the potential of directly examining treatment transcripts was shown in a study by Perez-Rosas et al. [55], where combined n-grams, lexicon, and linguistic features were as predictive of patient-rated quality as the use of behavioral codes (*F*_1_ = 0.87 with/out human annotations). Language models can also be used to generate treatment fidelity labels: Ewbank and colleagues [34] automatically segmented CBT sessions into intervention components (e.g., Socratic questioning), with varying degrees of accuracy (*F*_1_ = 0.22-0.94). They then showed how algorithmically identified CBT factors differentially increased the likelihood of engagement and symptom improvement (GAD-7 & PHQ-9 scores) for 17,572 patients.

##### Provider characteristics (*n* = 12)

*Empathy.* Seven studies focused on the assessment of empathy, given its role in establishing treatment alliance [16]. Early models assessed session-level empathy by examining behavioral codes [58, 59, 78, 124]. Contextual language models and larger datasets examined utterance-level empathy [68], also in specific expressive forms (i.e., reactions, interpretations, and explorations) with sufficient accuracy (EPITOME codes; *F*_1_ = 0.74) [62]. Similarly, Zhang and Danescu-Niculescu-Mizil [125] designed a model using 1.5 million crisis center conversations to identify whether providers responded to patients’ empathetically versus advancing toward concrete resolutions. In addition to measuring empathy, Transformer-based architectures have emerged as a tool for augmenting providers’ empathy, with one study using generative language models to suggest more empathic rewriting of text-based interventions [73]. *Conversational skills.* Five manuscripts examined the linguistic ability of providers [33, 50, 65, 125, 126]. One study [33] generated a model from 80,885 counseling interventions to extract therapist conversational factors, and showed how differences in content and timing of these factors predicted outcome (patient-reported helpfulness; AUC = 0.72). Importantly, conversational markers not only captured between-provider differences, but also found within-provider differences related to patients’ diagnoses [50] and as they gained clinical experience over time [65].

#### Patient-provider interaction analysis (n = 21)

##### Relational dynamics (*n* = 14)

*Therapeutic alliance and ruptures.* The study of patient-provider interactions primarily focused on analyzing therapeutic alliance, given its association with treatment outcomes [16]. Six studies sought to determine ratings of alliance strength [36, 46] or moments of rupture [127–130]. In one application, the NLP model detected patient ruptures unidentified by providers [129] and associated ruptures with decreases in emotional engagement between providers and patients [128]. *Mutual affect analysis.* Five interaction studies examined provider-therapist emotional convergence [131, 132] during the intervention, including defense mechanisms [133] and humor [45]. Tanana and colleagues examined a large corpus of therapy transcripts [102], and showed that an attention-based architecture captured therapists’ and patients’ valence interactions and their context more accurately (*K* = 0.48) than previous lexicon methods (*K* = 0.25 and *K* = 0.31) and human raters (*K* = 0.42). *Linguistic coordination.* Three studies focused on semantic similarity and linguistic coordination in therapeutic dyads given its association with positive outcomes [63, 134]. Researchers examined the association of linguistic coordination with affective behaviors across different treatment settings [134], and the role of linguistic synchrony in more effective interventions [135].

##### Conversation topics (*n* = 7)

Seven studies focused on identifying conversational themes emerging from treatment interactions [61, 136–138], including identifying functioning issues [139] and capturing conversational changes following the COVID-19 pandemic [72]. Imel et al. [140]. also showed how treatment topics accurately reflect differences in therapeutic approaches (Accuracy =0.87).

### Limitations of reviewed studies

#### Bias towards English

Included studies overwhelmingly featured NLP analyses of English transcripts. English was the only source of conversational data for 87.3% of studies (*n* = 89). Of the remaining 13 manuscripts, three were Dutch [60, 85, 101], three were Hebrew [108, 127, 129], two were Cantonese [105, 130], two were German [44, 141], and Italian [61], Mandarin [88], and Polish [51] were each analyzed in a single study. This lack of linguistic diversity poses important questions on whether findings from the examination of English conversations can be generalized to other languages.

#### Limited reproducibility

While we reported algorithm performance where available, studies used different types of ground truth (e.g., psychiatrist-assessed [51] vs self-reported [122] autism) and reported different evaluation metrics (e.g., F-scores vs ROC AUC), which did not allow for meaningful direct comparisons across all studies. The examined studies were also limited in their availability of open data and open code: only a fraction of studies made their computational code (*n* = 16; 15.7%) or their data (*n* = 8; 7.8%) available. Although several studies provided graphical representations of their model architecture [52], information on algorithmic implementation, model hyper-parameters, and random seeds were typically left under-specified. Five deep learning studies mitigated this limitation by utilizing an interpretability algorithm to elucidate their models [71, 79, 85, 104, 122]. While data unavailability is understandable given concerns for patient privacy and remains a significant challenge to future work, the absence of detailed model information, shared evaluation metrics, and code is a critical obstacle to the replication and extension of findings to new clinical populations.

#### Population bias

A third limitation was the lack of sample diversity. Data for the studies were predominantly gathered from the US. Moreover, the majority of studies didn’t offer information on patient characteristics, with only 40 studies (39.2%) reporting demographic information for their sample. In addition, while many studies examined the stability and accuracy of their findings through cross-validation and train/test split, only 4 used external validation samples [89, 107, 134] or an out-of-domain test [100]. In the absence of multiple and diverse training samples, it is not clear to what extent NLP models produced shortcut solutions based on unobserved factors from socioeconomic and cultural confounds in language [142].

## Discussion

In this systematic review we examined 102 applications of Natural Language Processing (NLP) for Mental Health Interventions (MHI) to evaluate their potential for informing research and practice on challenges experienced in the mental healthcare system. NLP methods are uniquely positioned to enhance language tasks with the potential to reduce provider burden, improve training and quality assurance, and more objectively operationalize MHI. To advance research in these areas, we highlight six clinical categories that emerged in the review. For the patient: (1) clinical presentation, including patient symptoms, suicide risk, and affect; and (2) intervention response, to monitor patient response during treatment. For the provider: (3) intervention monitoring, to evaluate the features of the administered treatment; and (4) provider characteristics, to study the person of the therapist and their conversational skills and traits. For patient-provider interactions: (5) relational dynamics, to evaluate alliance and relational coordination; and (6) conversational topics, to determine treatment content. In terms of language models, studies showed a shift from word count and frequency-based lexicon methods to context-sensitive deep neural networks. The growth of context-sensitive analyses appeared to follow increased prevalence of digital platforms and large corpora generated by telemedicine MHI. Acoustic features were another promising source of treatment data, although linguistic content was a richer source of information in the reviewed studies. Research in this area demonstrated progress in the areas of diagnostics, treatment specification, and the identification of contributors to outcome including the quality of the therapeutic relationship and markers of change for the patient. We propose integrating these disparate contributions into a single framework (NLPxMHI) to summarize promising avenues for increasing the utility of NLP for mental health service innovation.

### NLPxMHI research framework

**Fig. 4:**
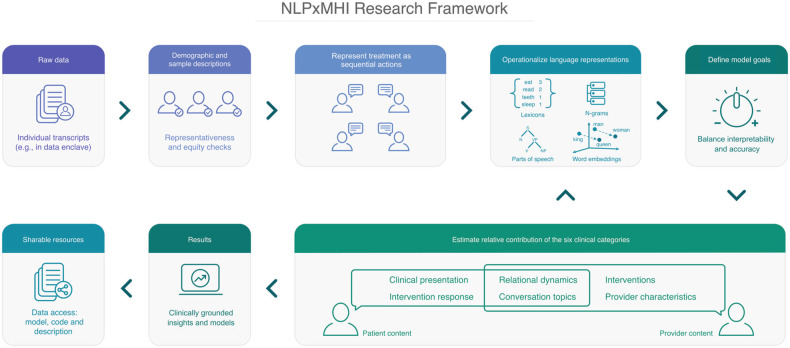
NLPXMHI Framework workflow. A MHI transcripts corpus is reviewed for its representativeness. If deemed appropriate for the intended setting, the corpus is segmented into sequences, and the chosen operationalizations of language are determined based on interpretability and accuracy goals. Model features for the six distinct clinical categories are designed. If necessary, investigators may adjust their operationalizations, model goals and features. If no changes are needed, investigators report results for clinical outcomes of interest, and support results with sharable resources including code and data.

The goal of the NLPxMHI framework (Fig. 4) is to facilitate interdisciplinary collaboration between computational and clinical researchers and practitioners in addressing opportunities offered by NLP. It also seeks to draw attention to a level of analysis that resides between micro-level computational research [44, 47, 74, 83, 143] and macro-level complex intervention research [144]. The first evolves too quickly to meaningfully review, and the latter pertains to concerns that extend beyond techniques of effective intervention, though both are critical to overall service provision and translational research. The process for developing and validating the NLPxMHI framework is detailed in the Supplementary Materials.

#### Demographic and sample descriptions for representativeness, fairness, and equity

Recent challenges in machine learning provide valuable insights into the collection and reporting of training data, highlighting the potential for harm if training sets are not well understood [145]. Since all machine learning tasks can fall prey to non-representative data [146], it is critical for NLPxMHI researchers to report demographic information for all individuals included in their models’ training and evaluation phases. As noted in the Limitations of Reviewed Studies section, only 40 of the reviewed papers directly reported demographic information for the dataset used. The goal of reporting demographic information is to ensure that models are adequately powered to provide reliable estimates for all individuals represented in a population where the model is deployed [147]. While the US-based population bias for papers in the review may not be easily overcome through expansion of international population data, US domestic research can reduce hidden population bias by reporting language-relevant demographic data for the samples studied, as such data may signal to other researchers’ findings influenced by dialect, geography, or a host of other factors. In addition to reporting demographic information, research designs may require over-sampling underrepresented groups until sufficient power is reached for reliable generalization to the broader population. Relatedly, and as noted in the Limitation of Reviewed Studies, English is vastly over-represented in textual data. There does appear to be growth in non-English corpora internationally and we are hopeful that this trend will continue. Within the US, there is also some growth in services delivered to non-English speaking populations via digital platforms, which may present a domestic opportunity for addressing the English bias.

There are additional generalizability concerns for data originating from large service providers including mental health systems, training clinics, and digital health clinics. These data are likely to be increasingly important given their size and ecological validity, but challenges include overreliance on particular populations and service-specific procedures and policies. Research using these data should report the steps taken to verify that observational data from large databases exhibit trends similar to those previously reported for the same kind of data. This practice will help flag whether particular service processes have had a significant impact on results. In partnership with data providers, the source of anomalies can then be identified to either remediate the dataset or to report and address data weaknesses appropriately. Another challenge when working with data derived from service organizations is data missingness. While imputation is a common solution [148], it is critical to ensure that individuals with missing covariate data are similar to the cases used to impute their data. One suggested procedure is to calculate the standardized mean difference (SMD) between the groups with and without missing data [149]. For groups that are not well-balanced, differences should be reported in the methods to quantify selection effects, especially if cases are removed due to data missingness.

#### Represent treatment as sequential actions

We recommend representing treatment as sequential actions taken by providers and patients, instead of aggregating data into timeless corpora, to reduce unnecessary noise, enhancing the precision of effect estimates for intervention studies [52, 71, 109]. The reviewed studies highlight the potential benefits of embedding textual units into time-delimited sequences [52]. Longitudinal designs, while admittedly more complex, can reveal dynamics in intervention timing, change, and individual differences [150], that are otherwise lost. For example, the relationship between a specific intervention and outcome is intricate, as timing and context are important moderators of beneficial effects [113, 114]. There are no universal rules for determining how to sequence data, however the most promising avenues are: (1) turn taking; (2) the span between outcome measures; (3) sessions; or (4) clinically meaningful events arising from within, or imposed from outside the treatment.

#### Operationalize language representations and estimate contribution of the six clinical categories

The systematic review identified six clinical categories important to intervention research for which successful NLP applications have been developed [151–155]. While each individually reflects a significant proof-of-concept application relevant to MHI, all operate simultaneously as factors in any treatment outcome. Integrating these categories into a unified model allows investigators to estimate each category’s independent contributions—a difficult task to accomplish in conventional MHI research [152]—increasing the richness of treatment recommendations. To successfully differentiate and recombine these clinical factors in an integrated model, however, each phenomenon within a clinical category must be operationalized at the level of utterances and separable from the rest. The reviewed studies have demonstrated that this level of definition is attainable for a wide range of clinical tasks [34, 50, 52, 54, 73]. Utterance-level operationalization exists for some therapy frameworks [153, 154], which can serve as exemplars to inform the specification process for other treatment approaches that have yet to tie aspects of speech to their proposed mechanisms of change and intervention. For example, it is not sufficient to hypothesize that cognitive distancing is an important factor of successful treatment. Researchers must also identify specific words in patient and provider speech that indicate the occurrence of cognitive distancing [112], and ideally just for cognitive distancing. This process is consonant with the essentials of construct and discriminant validity, with others potentially operative as well (e.g., predictive validity for markers of outcome, and convergent validity for related but complementary constructs). As research deepens in this area, we expect that there will be increasing opportunities for theory generation as certain speech elements, whether uncategorizable or derived through exploratory designs, remain outside of operationalized constructs of known theory.

#### Define model goals: interpretability and accuracy

Model interpretability is used to justify clinical decision-making and translate research findings into clinical policy [156]. However, there is a lack of consensus on the precise definition of interpretability and on the strategies to enhance it in the context of healthcare [157]. We suggest that enhancing interpretability through clinical review, model tuning, and generalizability is most likely to produce valid and trustworthy treatment decision rules [158] and to deliver on the personalization goals of precision medicine [159]. The reviewed studies show trade-offs between model performance and interpretability: lexicon and rule-based methods rely on predefined linguistic patterns, maximizing interpretability [33, 112], but they tend to be less accurate than deep learning models that account for more complex linguistic patterns and their context [71]. The interpretability of complex neural architectures, when deployed, should be improved at the *instance-level* to identify the words influencing model predictions. Methods include examining attention mechanisms, counterfactual explanations, and layer-wise relevance propagation. Surprisingly, only a handful of the reviewed studies implemented any of these techniques to enhance interpretability. Nevertheless, these methods don’t offer interpretation of the *overall* behavior of the model across all inputs. We expect that ongoing collaboration between clinical and computational domains will slowly fill in the gap between interpretability and accuracy through cyclical examination of model behavior and outputs. We also expect the current successes of large language models such as GPT-4 and LLaMa [160] to be further enhanced and made more clinically interpretable through training on data where relationships among the NLPxMHI categories and clinical outcomes is better understood. Meanwhile, the tradeoff between accuracy and interpretability should be determined based on research goals.

#### Results: clinically grounded insights and models

A sign of interpretability is the ability to take what was learned in a single study and investigate it in different contexts under different conditions. Single observational studies are insufficient on their own for generalizing findings [152, 161, 162]. Incorporating multiple research designs, such as naturalistic, experiments, and randomized trials to study a specific NLPxMHI finding [73, 163], is crucial to surface generalizable knowledge and establish its validity across multiple settings. A first step toward interpretability is to have models generate predictions from evidence-based and clinically grounded constructs. The reviewed studies showed sources of ground truth with heterogeneous levels of clinical interpretability (e.g., self-reported vs. clinician-based diagnosis) [51, 122], hindering comparative interpretation of their models. We recommend that models be trained using labels derived from standardized inter-rater reliability procedures from within the setting studied. Examples include structured diagnostic interviews, validated self-report measures, and existing treatment fidelity metrics such as MISC [67] codes. Predictions derived from such labels facilitate the interpretation of intermediary model representations and the comparison of model outputs with human understanding. Ad-hoc labels for a specific setting can be generated, as long as they are compared with existing validated clinical constructs. If complex treatment annotations are involved (e.g., empathy codes), we recommend providing training procedures and metrics evaluating the agreement between annotators (e.g., Cohen’s kappa). The absence of both emerged as a trend from the reviewed studies, highlighting the importance of reporting standards for annotations. Labels can also be generated by other models [34] as part of a NLP pipeline, as long as the labeling model is trained on clinically grounded constructs and human-algorithm agreement is evaluated for all labels.

Another barrier to cross-study comparison that emerged from our review is the variation in classification and model metrics reported. Consistently reporting all evaluation metrics available can help address this barrier. Modern approaches to causal inference also highlight the importance of utilizing expert judgment to ensure models are not susceptible to collider bias, unmeasured variables, and other validity concerns [155, 164]. A comprehensive discussion of these issues exceeds the scope of this review, but constitutes an important part of research programs in NLPxMHI [165, 166].

#### Sharable resources: data access

The most reliable route to achieving statistical power and representativeness is more data, which is challenging in healthcare given regulations for data confidentiality and ethical considerations of patient privacy. Technical solutions to leverage low resource clinical datasets include augmentation [70], out-of-domain pre-training [68, 70], and meta-learning [119, 143]. However, findings from our review suggest that these methods do not necessarily improve performance in clinical domains [68, 70] and, thus, do not substitute the need for large corpora. As noted, data from large service providers are critical for continued NLP progress, but privacy concerns require additional oversight and planning. Only a fraction of providers have agreed to release their data to the public, even when transcripts are de-identified, because the potential for re-identification of text data is greater than for quantitative data. One exception is the Alexander Street Press corpus, which is a large MHI dataset available upon request and with the appropriate library permissions. Access to richer datasets from current service providers typically require data use agreements that stipulate the extent of data use for researchers, as well as an agreement between patients and service providers for the use of their data for research purposes. While these practices ensure patient privacy and make NLPxMHI research feasible, alternatives have been explored. One such alternative is a data enclave where researchers are securely provided access to data, rather than distributing data to researchers under a data use agreement [167]. This approach gives the data provider more control over data access and data transmission and has demonstrated some success [168].

### Limitations

While this review highlights the potential of NLP for MHI and identifies promising avenues for future research, we note some limitations. Although study selection bias was limited by pre-registered review protocol and by inclusion of peer-reviewed conference papers, theoretical considerations suggest possible publication bias in the selection of the reported results toward positive findings (i.e., file-drawer effect). In particular, this might have affected the study of clinical outcomes based on classification without external validation. Moreover, included studies reported different types of model parameters and evaluation metrics even within the same category of interest. As a result, studies were not evaluated based on their quantitative performance. Future reviews and meta-analyses would be aided by more consistency in reporting model metrics. Lastly, we expect that important advancements will also come from areas outside of the mental health services domain, such as social media studies and electronic health records, which were not covered in this review. We focused on service provision research as an important area for mapping out advancements directly relevant to clinical care.

## Conclusions

NLP methods hold promise for the study of mental health interventions and for addressing systemic challenges. Studies to date offer a large set of proof-of-concept applications, highlighting the importance of clinical scientists for operationalizing treatment, and of computer scientists for developing methods that can capture the sequential and context-dependent nature of interventions. The NLPxMHI framework seeks to integrate essential research design and clinical category considerations into work seeking to understand the characteristics of patients, providers, and their relationships. Large secure datasets, a common language, and fairness and equity checks will support collaboration between clinicians and computer scientists. Bridging these disciplines is critical for continued progress in the application of NLP to mental health interventions, to potentially revolutionize the way we assess and treat mental health conditions.

### Supplementary information

Supplementary Materials

PRISMA checklist

## Data Availability

Data for the current study were sourced from reviewed articles referenced in this manuscript. Literature search string queries are available in the supplementary materials.
