# Supplementary material for: Natural language processing for mental health interventions: a systematic review and research framework
Source: Transl Psychiatry. 2023 Oct 6;13:309. doi: 10.1038/s41398-023-02592-2 (PMC10556019; doi:10.1038/s41398-023-02592-2)
Supplement: Supplementary file 1 — Supplementary Materials [file 41398_2023_2592_MOESM1_ESM.docx]

**S1. Search string queries**

***PUBMED***

Search string: (("psychotherapy"[Title/Abstract] OR "counseling"[Title/Abstract] OR "therapy"[Title/Abstract] OR "behavioral health"[Title/Abstract] OR “psychiatry”[Title/Abstract] OR “hotline”[Title/Abstract] OR "crisis line"[Title/Abstract] OR “mental health”[Title/Abstract]) AND ("natural language processing"[Title/Abstract] OR "machine learning"[Title/Abstract] OR "computational linguistics"[Title/Abstract] AND (english[Filter]))

Filters:

• Language: English

***SCOPUS***

Search string: ((TITLE-ABS-KEY ( "psychotherapy") OR TITLE-ABS-KEY (“counselling”) OR TITLE-ABS-KEY (“therapy”) OR TITLE-ABS-KEY (“psychiatry”) OR TITLE-ABS-KEY (“hotline”) OR TITLE-ABS-KEY (“crisis line”) OR TITLE-ABS-KEY (“mental health”)) AND (TITLE-ABS-KEY ( "natural language processing") OR TITLE-ABS-KEY ( "machine learning" OR TITLE-ABS-KEY ( "computational linguistics")) AND ( LIMIT-TO ( LANGUAGE , "English"))

Filters:

• Document type: Article

• Language: English

***PSYCINFO***

Search string: ((psychotherapy OR counseling OR therapy OR (behavioral health) OR psychiatry OR hotline OR (crisis line) OR (mental health)) AND ((natural language processing) OR (machine learning)))

Filters:

• Document type: Article

• Language: English

**Supplementary Figure 1.** Overview of the NLPxMHI framework development.


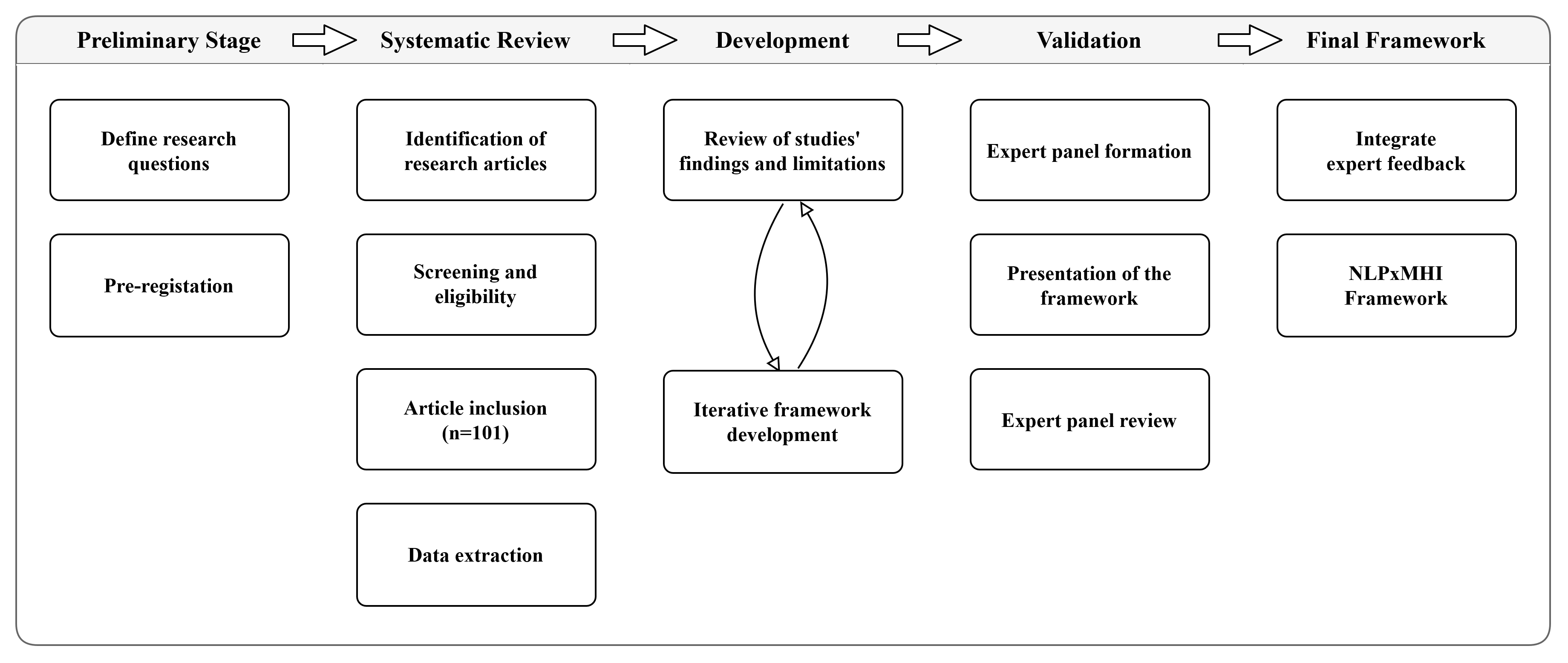


**S2. Research Framework Development**

A research framework is proposed for Natural Language Processing applications to mental health interventions (NLPxMHI) to improve their clinical interpretability, data access, and fairness. The NLPxMHI framework aims to facilitate interdisciplinary collaboration between computational and clinical domains of expertise, to further enhance future NLP applications and research in the mental health domains for MHI. The process of framework development consisted of a multi stage process to ensure comprehensibility and validity of its contribution. Framework development was divided in four steps, which included a thorough literature review, an iterative development with multiple revisions, and a validation by external experts (Supplementary figure 1). We provide below a description of each step below.

**Preliminary stage**

***Research Questions.*** The systematic review and framework development were informed by the following research questions (RQ):

- RQ1. *What natural language processing (NLP) algorithms and methods have been deployed to study mental health?*
- RQ2. *What are the aggregated findings from the reviewed studies?*
- RQ3. *What are the benefits and drawbacks of using NLP in the context of mental health? What are best practices recommendations for future studies?*

***Pre-registration.*** Search strategies and criteria of the systematic review were defined and then pre-registered according to the ProSysRev registration form.^1^ The systematic review protocol was published on the Open Science Framework^2^ (osf.io/s52jh).

**Systematic Review**

To inform the framework development, the authors conducted an extensive literature review adhering to the PRISMA guidelines.^3^

***Identification of research articles.*** Supplementary research section S1 reports the search string queries. The focus of the search was on NLP, rather than AI or machine learning methods as a whole. We did not include the term “artificial intelligence” since the review pre-process indicated the term added ~70,000 non-empirical entries. We instead included the term “machine learning” given its use to broadly refer to NLP research in the mental health literature.

**Screening and eligibility.** The search of all databases was first performed on August 1, 2021, and then updated with a second search on January 8, 2023. Candidate manuscripts were evaluated against the inclusion and exclusion criteria initially based on their abstract and then on the full-text independently by two authors (JMZ and MM).

*Inclusion Criteria:*

(1) Be an original empirical study.

(2) Article written in English.

(3) Article vetted through peer-review.

(4) The study must have focused on mental health intervention.

(5) The study analyzed text-data that was gathered from mental health intervention (e.g., transcripts, message logs).

*Exclusion Criteria:*

1. Study focused on human-computer interventions.
2. Study analyzed text-based data not derived from human-to-human interactions (i.e., medical records, clinician notes)
3. Study focused exclusively on social media platform content (e.g., Reddit).
4. Study focused on a population other than adults (18+).
5. Study did not analyze data using NLP.
6. Article was a book chapter, editorial article, or commentary.

***Article inclusion.*** The PRISMA^3^ diagram describing article inclusion process flow is presented in the main manuscript (Figure 1). Disagreement on the inclusion of an article or its clinical categorization was discussed with all the authors following full-text review. When more than one publication by the same authors used the same study aim and dataset, only the study with the most technical information and advanced model was included, with others classified as near duplicate and removed. Reasons for exclusion were recorded. The bibliographies of included articles were scanned for additional publications that met search criteria. The final sample consisted of 102 research manuscript focusing on NLP applications to MHI.

***Data extraction.*** The 102 manuscripts included were analyzed by two authors (MM and JMZ) to extract clinical and computational characteristics. Refer to Data extraction section of the main manuscript for further information on the specifics of the extracted clinical components (i.e., setting, aims, transcript source, clinical measures, ground truths and raters) and NLP components (i.e., linguistic representations and features, classification models, validation methods, and software packages) of interest for the systematic review.

**Development of the framework**

The systematic review provided empirical evidence into the state of the field and current study limitations, to serve as empirical elements to ground the research framework.

***Review of studies’ findings and limitations.*** For a summary of findings refer to the main manuscript in the Results sections, as well as Table 1, Figure 2, and Figure 3.

***Iterative framework development.*** The authors (TDH, MM, TA) drafted a preliminary framework based on the findings from the literature review. Several rounds of revisions of the framework draft were conducted, incorporating all author’s feedback to better address the unique challenges of NLP research applied to mental health interventions. During each iteration, the framework was assessed against the following criteria:^4,5^

1. The framework’s characteristics should be based on the findings emerging from the systematic review. The framework should current limitations and biases identified from the systematic review.
2. The framework should directly address the challenges of applying NLP methods for MHI research.
3. The framework should be adaptable to multiple goals (e.g., assessment and treatment), different intervention types, and across clinical settings (e.g., in person and digital health modalities).
4. The framework should encompass critical aspects of research design NLP applied to MHI. This includes, but is not limited to: how to operationalize clinical constructs using language representations; how to segment patient-provider transcripts; how to include interpretability and evaluation metrics; how to ensure data access.
5. The framework should focus on durable aspects of research design and should not be based on elements that need to be constantly updated, such as specific NLP algorithm recommendations.
6. The framework should account for inclusiveness, representativeness, and other ethical considerations based on limitations emerged from reviewed studies.
7. The framework should be easily understood by researchers and practitioners from both clinical and computational domains.

**Framework validation**

To ensure the validity of the framework, an external validation process was conducted.

***Expert panel formation.*** A group of independent experts were invited to review the framework. The selection of a group of diverse and representative experts was a crucial step in the validation process.^6^ Experts were identified in the fields of artificial intelligence, bioinformatics, clinical psychology, psychiatry, and NLP, including translational NLP research. They were contacted by email and invited to participate in the framework validation process. The final expert panel consisted of six members from multiple research academic institutions:

- Patricia Areán, PhD. Professor in Psychiatry & Behavioral Sciences at the University of Washington and licensed clinical psychologist (expertise in Clinical Psychology and Digital Health).
- Kyunghyun Cho, PhD. Associate professor of computer science and data science at New York University (expertise in Artificial Intelligence and NLP).
- Trevor Cohen, MBChB, PhD, FACMI. Professor in Biomedical Informatics and Medical Education at the University of Washington (expertise in NLP and bioinformatics).
- Adam S. Miner, PhD. Clinical Assistant Professor in Psychiatry and Behavioral Sciences at Stanford University and licensed clinical psychologist (translational NLP research expertise in Clinical Informatics, Psychology, and Epidemiology).
- Eric C. Nook, PhD. Assistant Professor in Psychology at Princeton University and licensed clinical psychologist (translational research expertise in NLP applied to Clinical Psychology).
- Naomi M. Simon, MD, Ms. Professor in Psychiatry at NYU Grossman School of Medicine (expertise in Psychiatry and treatment effectiveness research).

These external experts were not involved in the initial development process and provided an evaluation of the framework.^6^

***Presentation of the framework.*** The feedback from the expert panel was collected using individual semi-structured interviews format.^7^ Before the interview, the experts were offered the latest draft of the framework. Individual interviews were scheduled with each member of the experts panel. The interviews were conducted using a combination of video conferencing and in-person meetings, each lasting approximately 60 minutes. During the interview, the external experts were provided with a comprehensive overview the review findings. Each expert was presented with the goals of the study, and then underwent a semi-structured presentation of the framework based on the following elements:

- Objective and rationale of the framework.
- Clinical Categories (review of the clinical data sources identified and their focus).
- Limitations identified in the examined literature.
- Research Design (measurement operationalization, sequencing, and interpretable models).
- Data checks and reporting (best practices regarding sources of data, patients’ information, under represented individuals).
- Figures overviewing the NLPxMHI (i.e., alternative drafts of Figure 4).

***Expert panel review.*** The external experts were asked to critically assess the clinical categories identified and the proposed research framework, with the goal of providing revisions for improvement. They were asked to base their review on the same criteria used during the framework development stage (see Iterative framework development section). Each expert’s feedback on the semi-structured interview elements was transcribed and then aggregated. All experts acknowledged the transformative potential of applying NLP to mental healthcare interventions, and the importance of collaborations between NLP and mental health experts. All experts agreed on the identified limitations and biases emerging from the systematic review. The expert agreed on the clinical categories identified, and offered feedback on study categorization as well as meso-labels to better represent the emerging importance of patient-therapist interaction studies. The expert provided considerations and revisions regarding model accuracy, clinical interpretability, model interpretability, and the role of research aims in tuning the relative importance of accuracy and interpretability. The experts discussed the role of theory during the operationalization of clinical constructs. The experts also discussed challenges and opportunities to increase inclusiveness, linguistic diversity, and cultural representativeness in studies using NLP for MHI. The experts gave suggestions to improve clarity of the framework overview figure. The experts also provided additional scientific literature and existing reporting standards to support the framework.

**Final Framework**

***Integrate expert feedbacks.*** The framework was updated based on the aggregated feedback received by the external expert panel. The authors incorporated the experts' considerations into the framework and conducted additional revisions as needed. The revised framework was sent to the external experts once again, who then confirmed its validity.

***NLPxMHI Framework.*** The final framework, resulting from the iterative development process and external validation, is reported in the Discussion session of the main manuscript (NLPxMHI framework).

**References**

1. Van den Akker, O. *et al.* Inclusive systematic review registration form.

2. Foster, E. D. & Deardorff, A. Open science framework (OSF). *Journal of the Medical Library Association: JMLA* **105**, 203 (2017).

3. Page, M. J. *et al.* The PRISMA 2020 statement: an updated guideline for reporting systematic reviews. *International journal of surgery* **88**, 105906 (2021).

4. Saracevic, T. Relevance: A review of and a framework for the thinking on the notion in information science. *Journal of the American Society for information science* **26**, 321–343 (1975).

5. Gough, D. Weight of evidence: a framework for the appraisal of the quality and relevance of evidence. *Research papers in education* **22**, 213–228 (2007).

6. Okoli, C. & Pawlowski, S. D. The Delphi method as a research tool: an example, design considerations and applications. *Information & management* **42**, 15–29 (2004).

7. Bogner, A., Littig, B. & Menz, W. *Interviewing experts*. (Springer, 2009).
